# Targeting hepatic cholesterol sensing to tackle metabolic dysfunction–associated steatohepatitis

**DOI:** 10.1172/JCI206628

**Published:** 2026-07-15

**Authors:** Mengwei Zang, Yu Li

**Affiliations:** 1 Barshop Institute for Longevity and Aging Studies, Center for Healthy Aging, and; 2 Department of Molecular Medicine, University of Texas Health San Antonio, San Antonio, Texas, USA.; 3 Geriatric Research, Education and Clinical Center, South Texas Veterans Health Care System, San Antonio, Texas, USA.; 4 Shanghai Institute of Nutrition and Health, University of Chinese Academy of Sciences, Chinese Academy of Sciences, Shanghai, China.

## Abstract

Metabolic dysfunction–associated steatohepatitis (MASH) affects 1.5%–6.5% of the global population, yet its mechanisms remain incompletely understood. Cholesterol overload is a key driver of MASH, suggesting that targeting cholesterol sensing may offer therapeutic benefits. In this issue, Deng et al. identified nuclear factor erythroid 2–related factor 1 (NFE2L1) as a critical regulator linking cholesterol sensing to VLDL-mediated lipid export. Mechanistically, NFE2L1 interacts with insulin-induced gene 1 (INSIG1) and promotes its degradation in hepatocytes. This cholesterol-dependent NFE2L1-INSIG1 interaction sustains SREBP activation and VLDL secretion to maintain hepatic and systemic lipid homeostasis. Moreover, the study by Deng et al. indicates that hepatic NFE2L1 overexpression decreases INSIG1 abundance and ameliorates MASH progression, highlighting its therapeutic potential.

Metabolic dysfunction–associated steatotic liver disease (MASLD) is a global health crisis, spanning simple steatosis to steatohepatitis (metabolic dysfunction–associated steatohepatitis [MASH]) and cirrhosis (1, 2), with MASH affecting 1.5%–6.5% of the population (1, 2). However, its underlying mechanisms remain poorly understood. Accumulating evidence implicates chronic cholesterol overload as a key driver of MASH progression (3–6).

## Function of SREBPs on lipid metabolism

Sterol regulatory element–binding proteins (SREBPs), transcription factors in the endoplasmic reticulum (ER), form complexes with SREBP cleavage-activating protein (SCAP) and insulin-induced gene (INSIG) proteins (7–11). Under cholesterol-rich conditions, cholesterol binding to SCAP enhances the SCAP-INSIG1 interaction and stabilizes INSIG1, retaining the SCAP-SREBP complex in the ER and inhibiting Golgi trafficking and proteolytic processing of SREBP via negative feedback (9). SREBP1 activates the transcription of genes encoding enzymes involved in triglyceride synthesis, whereas SREBP2 regulates genes controlling cholesterol biosynthesis (7, 8). We have previously demonstrated that AMPK-mediated phosphorylation of SREBP inhibits its proteolytic cleavage, nuclear translocation, and transcriptional activation (12, 13), highlighting multilayered SREBP control. AMPK-dependent inhibition of SREBP may offer a therapeutic strategy to combat steatosis, insulin resistance, and atherosclerosis in obesity (12). However, the upstream regulators of INSIG1, particularly under cholesterol overload, remain elusive.

Although SREBP contributes to de novo lipogenesis and steatosis (14), emerging evidence suggests a nuanced role. Notably, hepatocytes continue to secrete VLDL under cholesterol-rich conditions, a compensatory process to prevent lipid accumulation (11, 15). Failure of this mechanism promotes hepatic lipid accumulation and activation of inflammatory and fibrogenic pathways, hallmarks of MASH. Yet, how VLDL secretion is maintained under cholesterol overload remains an important but unresolved question.

## Revealing the role of NFE2L1 in hepatic lipid homeostasis

**Figure 1:**
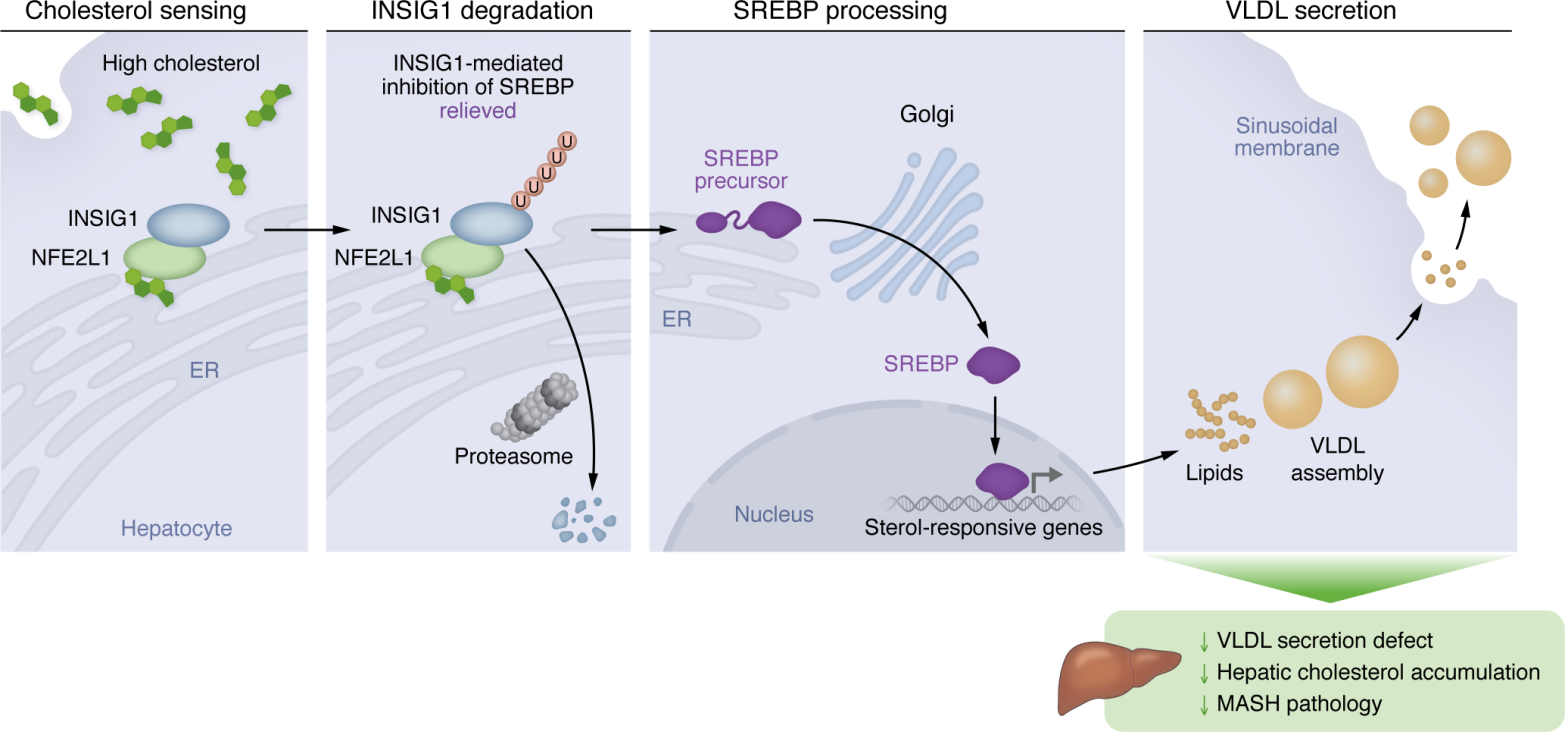
The NFE2L1/INSIG1 axis couples hepatic cholesterol sensing to VLDL secretion and maintains lipid homeostasis. Deng et al. (16) showed that under cholesterol overload, NFE2L1 acts as a sterol-responsive regulator that physically interacts with INSIG1 at the ER membrane in hepatocytes. This cholesterol-dependent interaction promotes ubiquitination and proteasomal degradation of INSIG1, relieving ER retention of SREBP precursors. Loss of INSIG1 permits ER-to-Golgi translocation of SREBP, where it undergoes proteolytic cleavage to generate the transcriptionally active mature form. The active SREBP enters the nucleus to induce transcription of sterol-responsive genes, enhancing cholesterol synthesis and lipogenesis. This structural lipid pool is functionally coupled to VLDL assembly and secretion, redirecting lipid flux from intracellular lipid synthesis to export. Newly synthesized lipids are incorporated into nascent VLDL particles and secreted via the sinusoidal membrane, preventing intracellular cholesterol accumulation. This adaptive process establishes a “synthesis-export balance,” in which the NFE2L1/INSIG1 axis synchronizes SREBP-mediated lipid synthesis with VLDL-mediated lipid export, thereby preventing hepatic cholesterol accumulation. By balancing lipid synthesis and export, the NFE2L1/INSIG1 axis attenuates VLDL secretion defects, limits intrahepatic cholesterol accumulation, and mitigates MASH progression, underscoring its therapeutic potential.

In this issue of the *JCI*, Deng et al. (16) have identified nuclear factor erythroid 2-related factor 1 (NFE2L1) as a critical regulator linking cholesterol sensing to VLDL-mediated export in hepatocytes (Figure 1). NFE2L1 is a transcription factor that regulates proteasome function and cellular antioxidative responses (17, 18), but Deng et al.’s study expands this role. Mechanistically, they report that NFE2L1 functions as a metabolic rheostat by interacting with INSIG1 and modulating its degradation in a cholesterol-dependent manner. In mice, hepatocyte-specific NFE2L1 deficiency diminished SREBP activity while simultaneously impairing VLDL assembly and secretion, thereby exacerbating cholesterol-driven lipotoxicity. Conversely, NFE2L1 overexpression reduced INSIG1 abundance and mitigated MASH pathogenesis by restoring lipid flux and balancing hepatic lipid biosynthesis with VLDL secretion. Given that NFE2L1 interplays with INSIG1 to coordinate lipid synthesis with VLDL secretion, this work highlights NFE2L1 as a previously underappreciated checkpoint in hepatocellular homeostasis and a potential therapeutic target for MASH (Figure 1).

## NFE2L1-INSIG1 interactions facilitate cholesterol-dependent INSIG1 degradation in hepatocytes

Prior to the study by Deng et al., work from the same research team demonstrated that NFE2L1 is an ER-resident protein whose localization, processing, and transcriptional activity are regulated by cholesterol in hepatocytes, establishing NFE2L1 as a cholesterol sensor (19). Here, Deng et al. (16) have elucidated how NFE2L1 interplays with INSIG1 in the regulation of hepatic and systemic cholesterol metabolism. Given their shared ER localization and sterol-sensing capabilities (9, 19), the authors hypothesized that NFE2L1 may regulate INSIG1 through direct interaction. Co-immunoprecipitation revealed that NFE2L1 was associated with INSIG1 in hepatocytes in a cholesterol-dependent manner via its 130 kDa ER-localized form, suggesting that this interaction occurs at the ER. Mechanistically, structure-function analyses revealed that the N-terminal homology box 2 (NHB2) domain of NFE2L1 was required for binding with INSIG1 but did not affect its subcellular localization or transcriptional activity. Importantly, free cholesterol enhanced the NFE2L1-INSIG1 interaction, supporting the notion that NFE2L1 functions as a cholesterol-sensitive regulator. This cholesterol-dependent NFE2L1-INSIG1 association negatively correlated with INSIG1 protein abundance. NFE2L1 promoted INSIG1 degradation predominantly via the ubiquitin-proteasome pathway. Consequently, loss of INSIG1 facilitated the proteolytic processing and activation of SREBP. Together, these findings uncover a previously unrecognized regulatory layer in which NHB2-mediated binding of NFE2L1 to INSIG1 links excessive cholesterol to INSIG1 degradation and SREBP activation (Figure 1).

## The NFE2L1/INSIG1 axis links cholesterol sensing to VLDL export

To assess the physiological significance of the NFE2L1-INSIG1 interaction, the authors used gain- and loss-of-function approaches. In hepatocytes, NFE2L1 knockdown reduced the active nuclear form of SREBP1 without affecting its precursor, indicating defective SREBP1 processing. In a fasting-refeeding model, liver-specific NFE2L1-knockout (LKO) mice exhibited reduced refeeding-induced nuclear SREBP1 and suppressed key lipogenic and cholesterogenic gene expression, consistent with observations in INSIG1-transgenic mice (9). Stable isotope tracing further revealed impaired de novo lipogenesis in NFE2L1-LKO livers. Interestingly, NFE2L1 deficiency reduced circulating cholesterol while increasing hepatic cholesterol content, indicating impaired lipid export. VLDL secretion assays further demonstrated reduced triglyceride export in NFE2L1-LKO mice. Mechanistically, ApoB release from hepatocytes was diminished by silencing NFE2L1 and rescued by constitutively active SREBP1, establishing that SREBP1 mediates the effect of NFE2L1 on VLDL secretion.

The authors further performed rescue experiments with AAV-mediated expression of WT NFE2L1 (NFE2L1-WT) or an INSIG1-binding–deficient (ΔNHB2) mutant in NFE2L1-LKO mice. Reconstitution with NFE2L1-WT, but not the ΔNHB2 mutant, restored SREBP1 activation in NFE2L1-LKO mice, indicating that INSIG1 mediates NFE2L1-driven SREBP1 signaling. Consistently, defective VLDL secretion in NFE2L1-LKO mice — as shown by reduced triglyceride export and decreased circulating ApoB and VLDL-cholesterol — was rescued by inducing expression of NFE2L1-WT but not the ΔNHB2 mutant, establishing that the NFE2L1/INSIG1 axis is essential for redirecting lipid flux from synthesis to VLDL secretion. Lipidomics profiling revealed reduced serum triglycerides and phosphatidylcholine — key components required for VLDL assembly — in NFE2L1-LKO mice, which were normalized by reintroducing NFE2L1-WT. Notably, NFE2L1-mediated inhibition of INSIG1 increased polyunsaturated fatty acids (PUFAs) (e.g., DHA) within triglyceride species. Collectively, the NFE2L1/INSIG1 axis acts as a key node linking cholesterol sensing to the balance between SREBP-mediated lipogenesis and VLDL secretion (Figure 1). Disruption of this adaptive mechanism in NFE2L1-LKO livers altered metabolic flux and reduced antiinflammatory PUFAs, thereby perturbing hepatic and systemic lipid homeostasis.

## The NFE2L1/INSIG1 axis protects against lipotoxicity and MASH

The transition from simple steatosis to MASH reflects a fundamental imbalance in metabolic pathways (3, 20). Although SREBP typically drives lipogenesis and lipid overproduction (8, 10, 20, 21), Deng et al. (16) emphasize that neither insufficient nor completely suppressed SREBP activity is beneficial, as basal SREBP activity is required to maintain lipid homeostasis by coupling lipid synthesis to VLDL-mediated export. Whereas the canonical model posits that cholesterol binding to SCAP promotes the SCAP-INSIG1 association and INSIG1 stabilization, inhibiting SREBP via negative feedback (8), the authors define a compensatory mechanism whereby NFE2L1 promotes INSIG1 degradation to sustain SREBP activity under chronic cholesterol overload. When SREBP-dependent synthesis of structural lipids essential for nascent VLDL biogenesis falls below a critical threshold, VLDL assembly becomes limiting. This impairs efficient packaging of triglycerides and cholesterol into VLDL particles, as observed in NFE2L1-LKO livers. The authors therefore propose that this process shunts lipid flux from secretion toward intracellular accumulation, exacerbating MASH. Thus, MASH may stem from both lipid overproduction and disrupted lipid trafficking and flux partitioning.

To test this hypothesis, the authors found that alongside impaired VLDL secretion, NFE2L1 deficiency evoked spontaneous liver injury and increased the expression of inflammatory and fibrotic genes. In contrast, adenoviral overexpression of NFE2L1 in *db/db* mice fed a methionine-choline–deficient (MCD) diet attenuated pathological features of MASH, including liver injury, lobular inflammation, and fibrosis. Mechanistically, NFE2L1 decreased INSIG1 abundance, enhanced SREBP1/-2 activation, and coordinated hepatic lipid synthesis with VLDL-mediated export. Similarly, statins — which are indirect activators of SREBP2 — improve MASH outcomes in humans (22, 23). Notably, NFE2L1 reduced hepatic cholesterol accumulation, while redistributing hepatic cholesterol into circulating antiatherogenic HDL, without increasing ApoB-containing lipoproteins. Although liver X receptor (LXR) has been implicated as a cholesterol sensor (24), NFE2L1 regulated cholesterol metabolism independently of LXR. Collectively, NFE2L1 functions as a pivotal hepatic cholesterol sensor that increases VLDL secretion and protects against MASH (Figure 1). This study further highlights a therapeutic strategy centered on redirecting metabolic flux from hepatic lipid synthesis toward secretion.

While this study compellingly links NFE2L1 to INSIG1 regulation, the precise mechanism underlying NFE2L1-mediated INSIG1 degradation remains unclear. Identifying the posttranslational modifications would yield mechanistic insight. Because the authors used the *db/db* mice with short-term MCD feeding, it is uncertain whether dysregulation of the NFE2L1/INSIG1 axis contributes to early MASLD, particularly in obesity-induced steatosis. Future studies with alternative MASH models and longitudinal human tissue analyses are vital to ascertain whether targeting this pathway can prevent disease onset and progression and establish its translational relevance. Given that cholesterol upregulates hepatocyte TAZ (WWTR1), a transcriptional coactivator, to drive fibrogenesis during MASH (5, 25), it will be important to determine whether hepatocyte NFE2L1 exerts paracrine effects that counteract fibrotic remodeling in MASH.

## Conclusion

Deng et al. have uncovered a previously unappreciated mechanism whereby the NFE2L1/INSIG1 axis couples cholesterol sensing to SREBP activation and VLDL secretion in hepatocytes (16). The NFE2L1-INSIG1 interplay orchestrates a critical balance between lipid synthesis and VLDL secretion to mitigate hepatic cholesterol accumulation and MASH in mice. While its clinical relevance remains to be established, this sterol-sensitive axis not only advances our understanding of lipid homeostasis but also offers a promising new target for therapeutic intervention in MASH, addressing a critical unmet need.

## Author contributions

MZ contributed to the financial support and writing of the manuscript. YL contributed to writing of the manuscript.

## Conflict of interest

The authors have declared that no conflict of interest exists.

## Funding support

This work is the result of NIH funding, in whole or in part, and is subject to the NIH Public Access Policy. Through acceptance of this federal funding, the NIH has been given a right to make the work publicly available in PubMed Central.

- NIH grants R01 AA031407, R01 DK100603, R01 DK121527, and T32 GM145432 (to MZ).
- Distinguished Chair Endowment Fund in Research from the Ewing Halsell Foundation at the University of Texas Health San Antonio (to MZ).

## Version 1 07/15/2026

Electronic publication
